# Early-onset kidney failure in a girl with autosomal dominant tubulointerstitial kidney disease due to a de novo *UMOD* variant

**DOI:** 10.1007/s13730-025-01081-3

**Published:** 2026-02-05

**Authors:** Shinya Tomori, Kenichiro Miura, Yoko Shirai, Taeko Hashimoto, Ichiro Hada, Ryota Kurayama, Naoya Morisada, Kandai Nozu, Motoshi Hattori

**Affiliations:** 1 Department of Pediatric Nephrology, Tokyo Women’s Medical University, 8-1 Kawada-cho, Shinjuku-ku, Tokyo, 162-0054 Japan; 2 Department of Pediatrics, Kyorin University Suginami Hospital, 2-25-1, Wada, Suginami-ku, Tokyo, 166-0012 Japan; 3 Department of Pediatrics, Kobe University Graduate School of Medicine, 7-5-1, Kusunoki-Cho, Chuo-Ku, Kobe, Hyogo 650-0017 Japan; 4 Kidney Disease Research Institute, Tokiwa Foundation, Jyoban Hospital, 57 Kaminodai, Joban Kamiyunagaya-machi, Iwaki-shi, Fukushima, 972-8322 Japan

**Keywords:** ADTKD-*UMOD*, Kidney failure, EGF-like domain

## Abstract

Autosomal dominant tubulointerstitial kidney disease (ADTKD) is characterized by renal tubular and interstitial abnormalities and slow progressive loss of kidney function. Patients with ADTKD rarely progress to kidney failure in early childhood. A 7-year-old Japanese girl was admitted to the hospital due to afebrile seizures and was later diagnosed with Panayiotopoulos syndrome. Blood examinations showed that she had a serum creatinine level of 1.57 mg/dL (Cr-eGFR 28 mL/min/1.73 m²), consistent with chronic kidney disease stage 4. Ultrasonography showed bilateral small to normal-sized kidneys, with increased renal parenchymal echogenicity, poor corticomedullary differentiation, and small cysts. A panel exome sequencing targeting 187 genes identified a de novo pathogenic variant c.172G > T, p.Gly58Cys in the EGF-like domain 1 of the *UMOD* gene. Her parents did not possess this variant, leading to the diagnosis of a sporadic case of ADTKD-*UMOD*. Variants in the EGF-like domain 1 may lead to early progression to kidney failure. ADTKD-*UMOD* should be listed as a differential diagnosis of progressive kidney failure in early childhood, even in the absence of a family history.

## Introduction

Autosomal dominant tubulointerstitial kidney disease due to *UMOD* variants (ADTKD-*UMOD*) is a hereditary nephropathy caused by pathogenic variants in the *UMOD* gene, which encodes the glycoprotein uromodulin and is located on chromosome 16p12. Clinically, it is characterized by chronic kidney disease (CKD) and hyperuricemia [1]. CKD in ADTKD-*UMOD* typically progresses during adulthood, with a reported median age at diagnosis of 30.5 years [2] and a median age at onset of kidney failure of 47 years [3].While there have been reports of ADTKD identified during childhood through family screening at early stages of CKD, progression to kidney failure during childhood is rare [1]. Here, we report a sporadic pediatric case of ADTKD-*UMOD* diagnosed during the evaluation of advanced CKD.

Case Presentation.

A 7-year-old girl, born to healthy, non-consanguineous Japanese parents, was brought to the emergency department for afebrile seizure. The seizure resolved spontaneously, and brain MRI and EEG revealed no abnormalities. Subsequently, the seizures were diagnosed as Panayiotopoulos syndrome. As an incidental finding, her serum creatinine level was elevated at 1.57 mg/dL (creatinine-based eGFR: 28 mL/min/1.73 m²), consistent with CKD stage 4. She was referred to our hospital for further evaluation.

She had no history of abnormal urinalysis findings, and no family history of kidney disease or dialysis. Her height was 115 cm (–0.6 SD) and weight was 18.5 kg (–1.0 SD). No abnormalities were found on physical examination. Laboratory tests showed a creatinine-based eGFR of 26 mL/min/1.73 m², cystatin C-based eGFR of 31 mL/min/1.73 m², hemoglobin 9.3 g/dL, and uric acid 9.3 mg/dL. Early morning urine showed a specific gravity of 1.006, indicating hyposthenuria.

Ultrasonography indicated that the left kidney measured 6.3 × 3.9 cm (–2.3 SD) and the right kidney measured 6.9 × 3.9 cm (–1.6 SD), suggesting that the kidneys were small to normal in size. Increased echogenicity and the presence of several small cysts were also observed. These findings were suggestive of nephronophthisis. In addition, punctate echogenic foci consistent with calcifications were observed within the parenchyma. No abnormalities were found in the bladder, ureters, liver, or other organs. Otolaryngologic and ophthalmologic evaluations revealed no extrarenal manifestations.

**Fig. 1:**
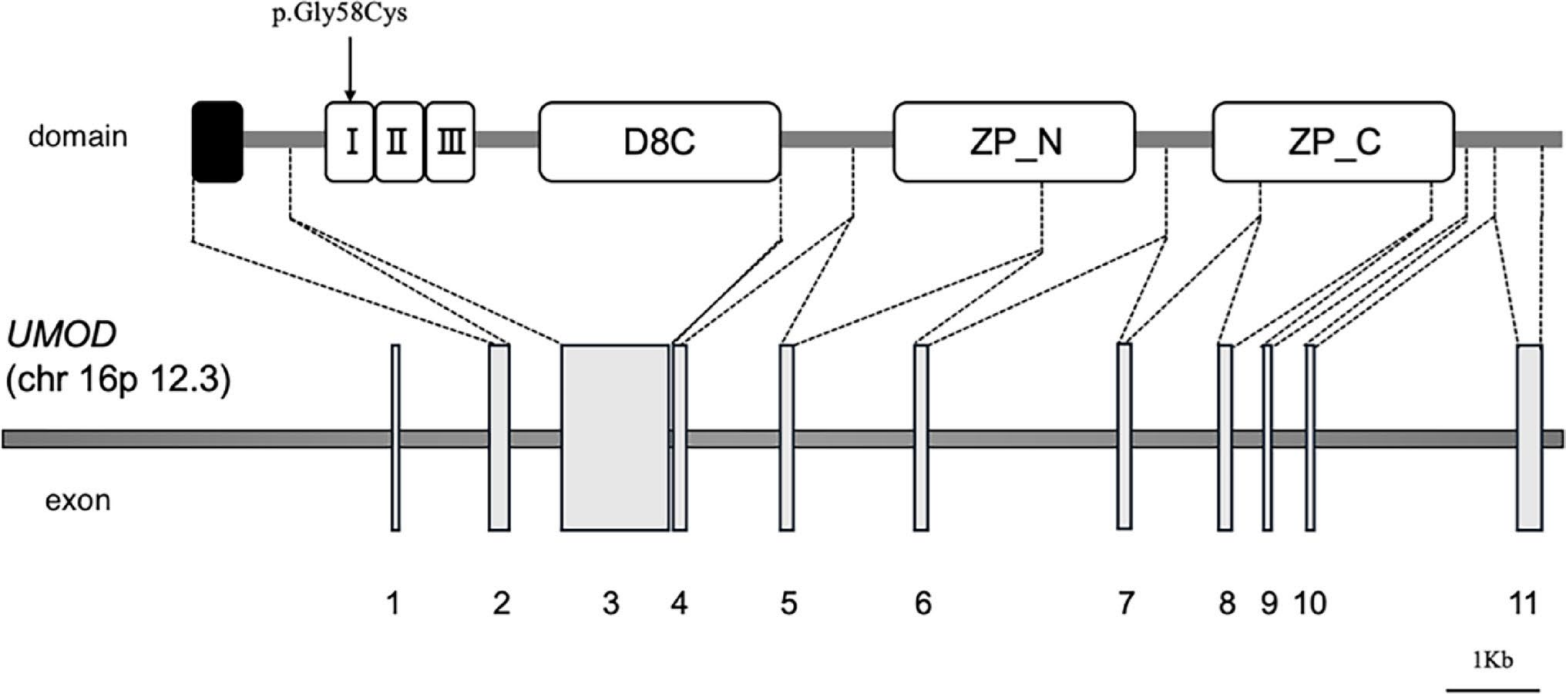
Schematic representation of the *UMOD* gene structure and corresponding protein domains The UMOD protein, which consists of three epidermal growth factor (EGF)–like domains, a D8C domain, and a zona pellucida (ZP) domain. The variant identified in this case, p.Gly58Cys (arrow), is located within EGF-like domain 1, which is encoded by exon 3 I, EGF-like domain 1; II, EGF-like domain 2; III, EGF-like domain 3; D8C, central domain; ZP_N, N-terminal subdomain of the zona pellucida domain; ZP_C, C-terminal subdomain of the zona pellucida domain

Due to the sporadic nature of the case and the presence of normal to small-sized kidneys with cysts and progressive CKD in childhood, nephronophthisis was initially suspected. However, genetic testing for nephronophthisis-related genes, conducted with parental consent, revealed no pathogenic variants. Subsequently, targeted panel exome analysis covering 187 genes related to congenital anomalies of the kidney and urinary tract (CAKUT) and cystic kidney diseases was performed in the proband. Sanger sequencing was then conducted for the patient and both parents, and the variant was detected only in the patient but not in the parents. This confirmed that it was a de novo variant, c.172G > T, p.Gly58Cys, in the EGF-like domain 1 of the *UMOD* gene (Fig. 1) [4]. Neither parent carried this variant. Based on the results of genetic analysis, the patient was diagnosed with ADTKD-*UMOD*. Treatment for CKD was initiated with oral sodium bicarbonate and febuxostat. Subsequent monitoring of renal function revealed a decline in eGFR to 19 mL/min/1.73 m² 1 year and 2 months after the initial visit to our hospital, at which point the patient was registered for deceased donor kidney transplantation.

## Discussion

We present a rare pediatric sporadic case of ADTKD-*UMOD* with a de novo pathogenic variant in the EGF-like domain 1, associated with advanced CKD at an earlier age than previously reported. ADTKD-*UMOD* is typically characterized by CKD progression during adulthood. Bleyer et al. reported that among 34 patients with ADTKD-UMOD under the age of 18, none had renal dysfunction corresponding to CKD stage 4 or higher [1]. However, there have been reports of pediatric patients progressing to kidney failure [1, 4, 5]. This case adds to the literature suggesting that although rare, ADTKD-*UMOD* may present with kidney failure during childhood.

ADTKD is typically characterized by a positive family history, which is also reflected in the diagnostic criteria [6]. However, approximately 10% of ADTKD-*UMOD* cases occur without a family history [7]. Our case lacked a family history, and initial differential diagnoses included CAKUT and nephronophthisis. Genetic testing ultimately confirmed a de novo pathogenic *UMOD* variant. Therefore, even in the absence of a family history, ADTKD-*UMOD* should be considered in the differential diagnosis of pediatric advanced CKD.

Uromodulin consists of EGF-like domains, a central D8C domain, a zona pellucida domain, and a GPI anchor [8]. Although the mechanism by which *UMOD* variantscause kidney disease remains unclear, EGF-like domains are common protein motifs involved in cell adhesion, coagulation, and receptor-ligand interactions. Moskowitz et al. reported that patients with variants in EGF-like domains 2 or 3 progressed to kidney failure at a younger age than those with variants in other domains [9]. Zaucke et al. reported two patients with the same variant as in our case, both progressing to severe CKD by age 12 and 13, respectively (eGFR 3 and 23 mL/min/1.73 m²) [4]. To date, five cases of pediatric-onset kidney failure due to ADTKD-*UMOD* have been reported (Table 1) [1, 4, 5], with a median age of kidney failure onset at 12 years. Of these, three had variants in EGF-like domain 1. These findings suggest that variants in the EGF-like domain may be associated with early-onset kidney failure.

**Table 1:** Previously reported cases of ADTKD-*UMOD* with kidney failure during childhood

No.	Author, Year	Age at Kidney Failure	Sex	Family History	Nucleotide variant	The change of amino acid sequence	Domain
1–1	Zaucke, 2010 [4]	13	N/A	Present (details unknown)	c.172G > T	p.Gly58Cys	EGF1
1–2		12	N/A	Present (details unknown)	c.172G > T	p.Gly58Cys	EGF1
2	Carucci, 2019 [5]	6	Male	Mother had the same variant (kidney failure at 31 years)	c.249 C > G	p.Cys83Trp	EGF2
3 − 1	Bleyer, 2022 [1]	12	N/A	Relatives had kidney failure at ages 18 and 24 (details unknown)	c.155G > C	p.Cys52Ser	EGF1
3 − 2		14	N/A		c.155G > C	p.Cys52Ser	EGF1

The median age of kidney failure among these five cases was 12 years, and four out of the five patients had variants in the EGF-like domain 1

## Conclusion

We report a sporadic pediatric case of ADTKD-*UMOD* identified through evaluation of advanced CKD. Although kidney failure during childhood is uncommon in ADTKD-*UMOD*, our case and previous reports indicate that variants in specific domains, such as the EGF-like domain, may lead to early disease progression. ADTKD-*UMOD* should be considered in the differential diagnosis of early-onset CKD and kidney failure in pediatric patients.
